# Clinical and microbiologic efficacy of the piperazine-based drug lead MMV665917 in the dairy calf cryptosporidiosis model

**DOI:** 10.1371/journal.pntd.0006183

**Published:** 2018-01-08

**Authors:** Erin Stebbins, Rajiv S. Jumani, Connor Klopfer, John Barlow, Peter Miller, Mary A. Campbell, Marvin J. Meyers, David W. Griggs, Christopher D. Huston

**Affiliations:** 1 Department of Medicine, University of Vermont Larner College of Medicine, Burlington, Vermont; 2 Cell, Molecular and Biomedical Sciences graduate program, University of Vermont Larner College of Medicine, Burlington, Vermont; 3 Department of Animal and Veterinary Sciences, University of Vermont College of Agriculture and Life Sciences, Burlington, Vermont; 4 Saint Louis University School of Medicine, St. Louis, MO; 5 Department of Microbiology and Molecular Genetics, University of Vermont Larner College of Medicine, Burlington, Vermont; Johns Hopkins Bloomberg School of Public Health, UNITED STATES

## Abstract

Cryptosporidiosis causes life-threatening diarrhea in infants, but the best available treatment is only modestly efficacious. Rodents infected with relevant *Cryptosporidium* species do not develop diarrhea, which complicates drug development. *Cryptosporidium parvum* infection of dairy calves, however, causes an illness like that seen in infants. Here, the clinical and microbiologic anti-*Cryptosporidium* efficacy of the piperazine-based compound MMV665917 was demonstrated in neonatal calves. Oral administration of MMV665917 (22 mg/kg once daily) was begun two days after the onset of severe diarrhea and continued for seven days. Treatment resulted in prompt resolution of diarrhea, and reduced total fecal oocyst shedding by ~94%. MMV665917 was useful for treatment, rather than just prophylaxis, since it was safe and effective when administered well after the onset of diarrhea. Furthermore, even though all animals received intensive supportive care, there was a strong trend towards improved secondary health outcomes, including general health, appetite, and dehydration measures amongst treated animals. These data establish MMV665917 as an outstanding lead compound for *Cryptosporidium* drug development.

## Author summary

Cryptosporidiosis is an important cause of life-threatening diarrhea for young children and immunocompromised people, and *Cryptosporidium parvum*, one of the two main human *Cryptosporidium* pathogens, is also an important cause of diarrhea in dairy calves. Yet, there are no reliably effective drugs for treating cryptosporidiosis. Anti-*Cryptosporidium* drug development is complicated by the fact that infected rodents do not develop diarrhea. Here, a dairy calf model of cryptosporidiosis was used to demonstrate that the piperazine-based compound MMV665917, a compound identified within the open-access Medicines for Malaria Venture “Malaria Box”, reduces both diarrhea and parasite shedding when given once daily for seven days to dairy calves infected with *C*. *parvum*. These data establish MMV665917 as one of just several compounds with proven efficacy for treating dairy calves with cryptosporidiosis, and an outstanding lead to develop for humans.

## Introduction

Diarrhea still causes over 10% of childhood deaths [1]. Although cryptosporidiosis was previously recognized as a major contributor to diarrhea in endemic areas, a recent multicenter study in Africa and Asia found that *Cryptosporidium* infections, mostly due to *Cryptosporidium hominis* or *Cryptosporidium parvum*, are the third most common cause of severe diarrhea in young children [2]. *Cryptosporidium* infections are also strongly associated with malnutrition and delayed development [2]. Furthermore, cryptosporidiosis is a prevalent cause of chronic diarrhea in AIDS patients [3], and is responsible for over 85% of waterborne diarrhea outbreaks in the United States [4].

Treatments for cryptosporidiosis in the most affected populations are poor. Nitazoxanide is the only drug with proven efficacy in immunocompetent people, in whom it shortens the illness by approximately one day. Nitazoxanide is equivalent to a placebo for HIV positive people, and data from very young, malnourished children come from a study of only 47 children; 14/25 (56%) nitazoxanide recipients were improved at seven days, while 5/22 (23%) placebo recipients were improved [5, 6]. There is a dire need to develop more effective treatments for people with cryptosporidiosis.

*Cryptosporidium parvum* infection in calves is an economic concern for beef and milk producers, and may contribute to contamination of water supplies and human disease outbreaks. In a nationwide survey of 1103 US farms, *Cryptosporidium* was present on over 50% of farms, infecting 48% of all calves aged 1–3 weeks [7]. Although most outbreaks of human cryptosporidiosis are due to anthroponotic transmission of human restricted isolates, zoonotic transmission of livestock associated isolates has caused large outbreaks [8]. Controlling cryptosporidiosis in calves would therefore be of economic benefit and reduce the burden of human disease.

We and others previously used a cell-based assay to identify potential *Cryptosporidium* drug leads [9–12], in combination with follow-up *in vivo* studies in immunocompromised mice. One of the most promising compounds found to date, MMV665917, was identified within the Medicines for Malaria Venture “Malaria Box”, an open access collection of 400 compounds with activity against the erythrocyte stages of *Plasmodium falciparum* [10, 13]. MMV665917 is a piperazine-based compound with high selectivity for the blood stages of malaria parasites and for *Cryptosporidium* species. Its potency is roughly equivalent for multiple *C*. *parvum* lab and field isolates, and for the *C*. *hominis* TU502 isolate, and MMV665917 treatment reduces parasite shedding to below detectable limits in a highly immunocompromised mouse model of chronic *C*. *parvum* infection (Jumani, et al. Submitted manuscript). A limitation of the existing mouse cryptosporidiosis models, however, is that infected mice do not develop diarrhea. Thus, while MMV665917 has proven microbiologic efficacy in immunocompromised mice, its clinical efficacy for treating cryptosporidiosis remains unknown.

Neonatal calves infected with *C*. *parvum* shed oocysts at high levels and develop diarrhea similar to that seen in young children, providing a clinical model for drug testing [14]. This study’s purpose was to determine if MMV665917 is both clinically and microbiologically efficacious in dairy calves. We report pharmacokinetic (PK) data from uninfected calves and infected calves with diarrhea, and the results of a clinical efficacy study.

## Methods

### Dairy calf clinical model of cryptosporidiosis

Animal studies were approved by the University of Vermont (UVM) Institutional Animal Care and Use Committee (IACUC). The University of Vermont has an Animal Welfare Assurance with the Office of Laboratory Animal Welfare (OLAW) of the National Institutes of Health, and is registered as a Research Institution by the United States Department of Agriculture. The University complies with the recommendations of the Guide for the Care and Use of Laboratory Animals (8th ed., NRC 2011) and with the Animal Welfare Act and its associated regulations (USDA-APHIS "Blue Book," available at www.aphis.usda.gov/animal-welfare). Holstein bull calves were acquired at birth from Green Mountain Dairy (Sheldon, VT), given synthetic colostrum with 200g of IgG (Land O’Lakes, Ardent Hills, MO) and bovine coronavirus and *Escherichia coli* antibodies (First Defense Bolus, Immucell Corporation, Portland, ME) within two hours of birth, and transported to UVM. Uninfected animals were group-housed in a pen for PK studies. For studies of infected calves, animals were initially group-housed and infected at 24–48 hours of age during an interruption in bottle feeding by oral administration of ~5×10^7^ viable *C*. *parvum* Iowa isolate oocysts (Bunch Grass Farms, Deary, ID) suspended in 10 mL of deionized water. Animals were moved to individual raised pens immediately after infection, and observed twice daily at feeding times for clinical signs, which were quantified according to a standardized scoring rubric (Table 1); fecal consistency, general health, hydration status, and appetite data were collected. Clinical microbiologic studies for adventitious infectious agents including *Salmonella* culture, aerobic bacterial culture with *E*. *coli* genotyping, and rotavirus and coronavirus testing were performed on all calves at the onset of diarrhea at the Cornell Health Diagnostic Center (Ithaca, NY). Animals with severe diarrhea and other symptoms were supported aggressively, including administration of oral electrolytes, intravenous fluids, and flunixin meglumine (Banamine, Merck) as needed. MMV665917 was suspended for dosing in 1% hydroxypropyl methyl-cellulose in water at a final volume of 10 mL per dose. Doses were squirted into the calves’ mouths during interruptions in bottle feeding. For PK studies, fecal samples were collected at the indicated times by manual anal stimulation. For treatment efficacy studies, daily fecal samples were obtained from collection bins located under each pen. Fecal samples used for parasite quantification were dried at 90°C until a stable weight was reached, and *C*. *parvum* abundance per gram of fecal dry matter was measured using a previously validated qPCR assay [15]. To our knowledge, this qPCR assay is the most sensitive method currently available. The lower limit of detection is ~100 oocysts/gram of dried feces.

**Table 1:** Criteria used for scoring clinical signs and symptoms, including fecal consistency, overall health, hydration, and appetite.

Score	Fecal Consistency	Health Status	Hydration Status	Appetite
**1**	Normal feces; feces retain form. The feces may be pasty but do not flow across a surface.	Normal: alert, hungry, interacts with caregivers	Normal; skin tents <1 second; moist mucus membranes	Normal: interacts with caregivers and eats greedily; eats 75–100% of meal
**2**	Mild-to-moderate diarrhea: unformed feces, flows down a surface, while leaving some residual	Mildly depressed: some loss of interest in feeding, equivocal hydration status	Mildly dehydrated: skin tents 1–4 seconds; normal mucus membranes	Mild inappetence: some loss of interest but eats 25–75% of meal
**3**	Severe diarrhea: part or all of feces is very watery; flows down a surface, while leaving no residual	Severely depressed: lethargic, must be coaxed to get up, anorexia, requires supportive treatment	Severely Dehydrated: skin won’t flatten when tented, eyes sunken, dry mucous membranes	Anorexic: loss of interest in feeding; eats 0–25% of meal

### Pharmacologic methods

Serum samples were analyzed by liquid chromatography-tandem mass spectrometry (LC/MS/MS), using compound spiked into control serum as a standard. Fecal MMV665917 was measured by homogenizing feces in PBS (0.1 g/mL) in a polypropylene tube and then further dilution prior to addition of an internal standard (enalapril) and acetonitrile protein precipitation. The supernatant was transferred to a fresh tube and dried using a speed vac. Samples were then resuspended and analyzed using LC/MS/MS.

### Data analysis, statistical methods, and figure preparation

Data were analyzed using GraphPad Prism version 6.00. The area under the curve (AUC) was calculated for each animal using a plot of the indicated parameter vs. time and a baseline score of one (i.e. a calf with a score of one every day (no diarrhea) would have an AUC of 0). The AUC for oocyst shedding was determined from a plot of the Log_10_ transformed fecal oocyst shedding per gram of fecal dry matter vs. time using a baseline of 2 (Log_10_100) and including the first day of drug dosing. p values were determined using the unpaired one-way student’s t test. Graphs were labeled for Fig preparation using Adobe Illustrator CS5.

## Results

### Pharmacokinetics of MMV665917 in dairy calves

**Fig 1:**
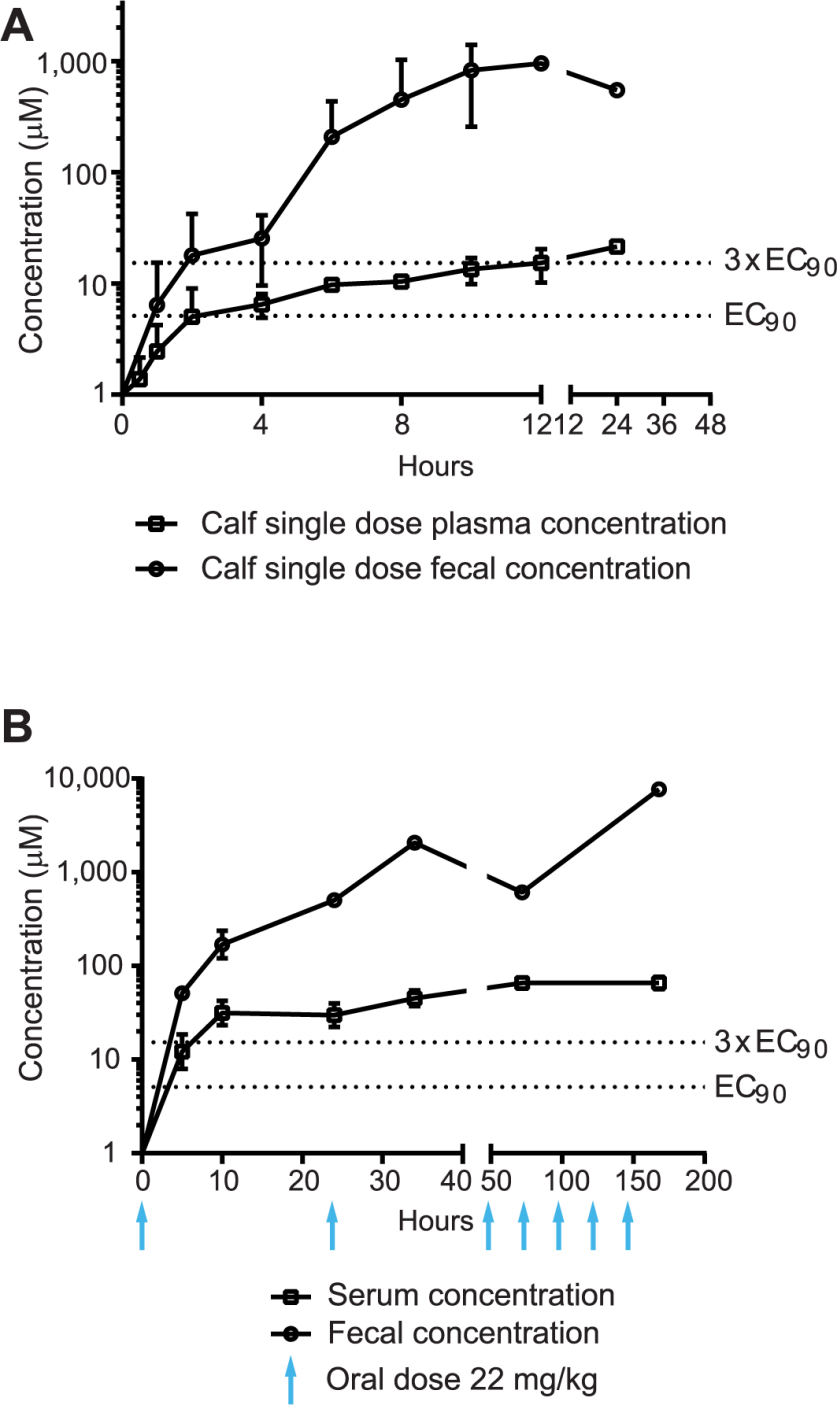
Pharmacokinetics of MMV665917 in dairy calves. **(A)** Uninfected calves. The graph shows calf fecal (circles) and serum (squares) MMV665917 concentrations vs. time following a single oral dose of 22 mg/kg given to healthy newborn calves. Data points are the means and SDs (n = 2 calves). **(B)** *Cryptosporidium parvum* infected calves. The graph shows calf fecal (circles) and serum (squares) MMV665917 concentrations vs. time for calves administered 22 mg/kg once daily for seven days, beginning 4 days after infection with the *C*. *parvum* Iowa isolate (indicated by blue arrows). The mean and SD (n = 3 calves) are shown.

It remains unknown if both intestinal and systemic compound concentrations are required for *in vivo* efficacy, but achieving a total sustained serum concentration of 3× the EC_90_ in an immunocompromised mouse model previously reduced parasites to below detectable levels, while mice treated at lower doses quickly relapsed (Jumani, et al. Submitted manuscript). To determine a comparable MMV665917 dosing regimen for clinical efficacy studies in the calf model, we therefore began with an experiment in which uninfected animals received a single oral dose, followed by measurement of serum and fecal compound concentrations (Fig 1A). The total MMV665917 serum concentration exceeded the target concentration of 3× the EC_90_ within 12 hours after receipt of 22 mg/kg orally, and this level persisted for over 24 hours. The fecal concentration of MMV665917 exceeded 3× the EC_90_ after just two hours, and persisted for over 24 hours. These data suggested the possibility of efficacy using a once daily dose of 22 mg/kg.

Because it remained possible that diarrhea in *Cryptosporidium* infected calves would alter the PK profile of MMV665917, we conducted a pilot study in infected animals using 22 mg/kg once daily administered for seven days beginning on day four post-infection, two days after the onset of severe diarrhea (Fig 1B). This dose resulted in sustained fecal and serum concentrations in excess of the target concentration. MMV665917 also appeared to be safe and well tolerated at this dose, since no adverse effects or taste aversion were noted.

### Clinical and microbiologic efficacy of MMV665917

**Fig 2:**
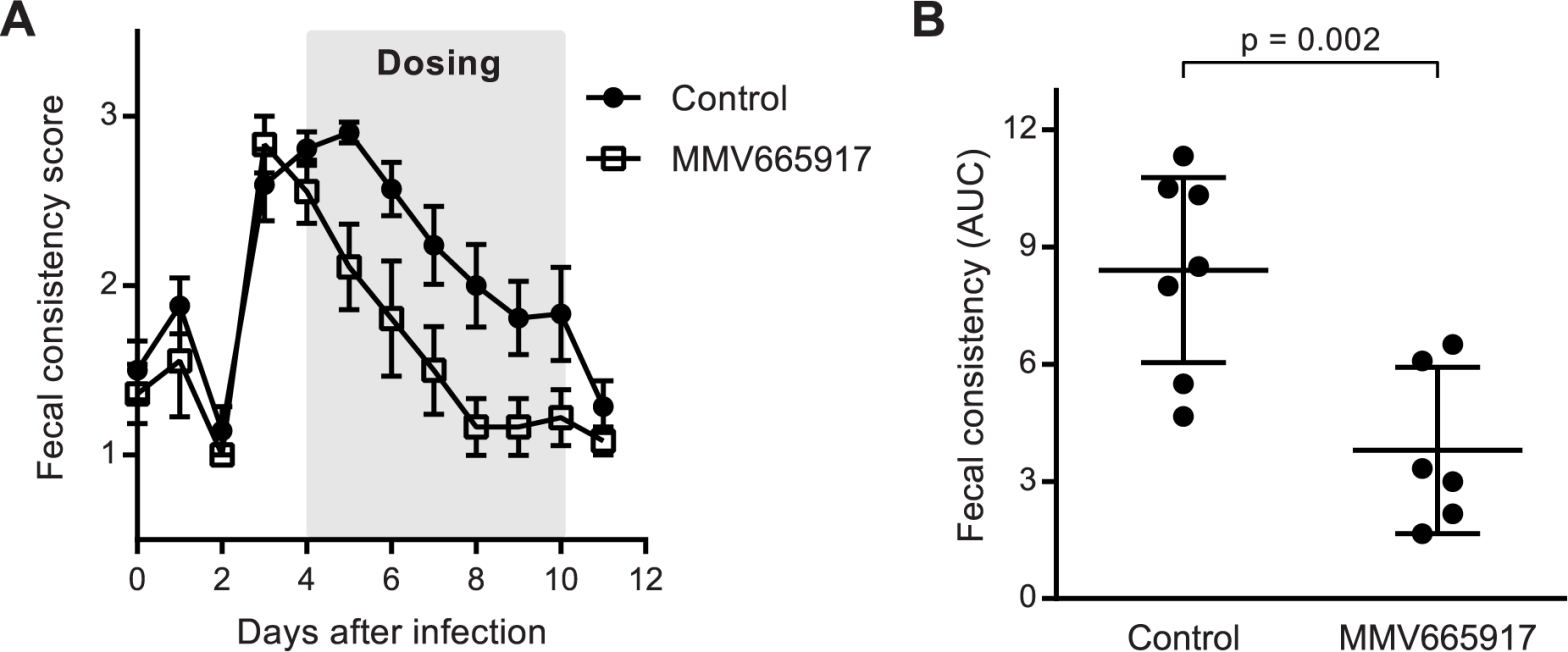
Once daily MMV665917 reduces diarrhea in *C*. *parvum* infected calves. Newborn dairy calves were infected with the *C*. *parvum* Iowa isolate and treated orally with either MMV665917 22 mg/kg once daily or drug vehicle alone from days 4–10 after infection, following development of severe diarrhea. Diarrhea was scored based on fecal consistency, with scores of 3 and 1 corresponding to severe diarrhea and normal, respectively. **(A)** Diarrhea scores vs. time for control (black circles) or MMV665917 treated (open squares) calves. Data are the means and SEMs (n = 7 for control; n = 6 for MMV665917 treated). **(B)** Scatter plot showing the area under the curve (AUC) for fecal score vs. time for individual animals. The lines indicate the means and 95% confidence intervals. p value was determined using the unpaired, single-sided student’s t test.

**Fig 3:**
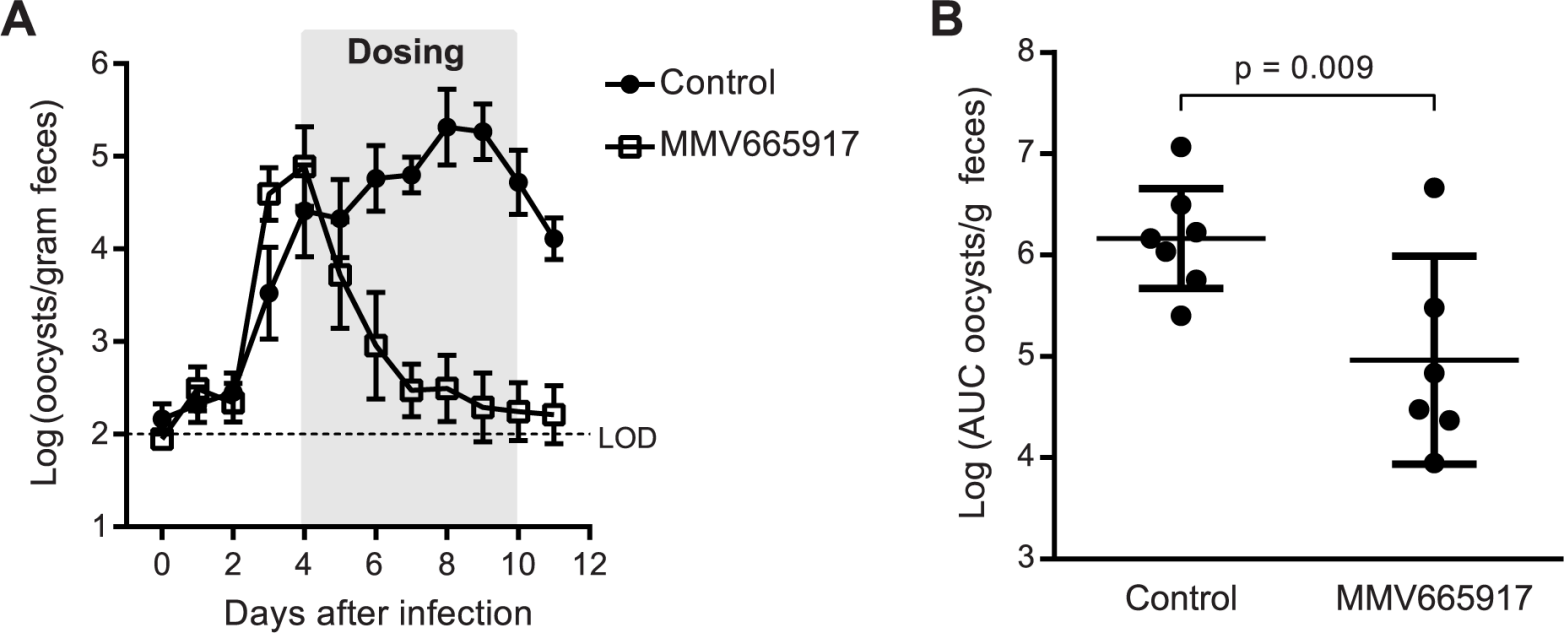
Once daily MMV665917 reduces fecal parasite shedding in *C*. *parvum* infected calves. Newborn dairy calves were infected with the *C*. *parvum* Iowa isolate and treated orally with either MMV665917 22 mg/kg once daily or drug vehicle alone from days 4–10 after infection, following development of severe diarrhea. Fecal oocyst shedding was measured using qPCR. **(A)** Fecal oocyst shedding vs. time for control (black circles) or MMV665917 treated (open squares) calves. Data are the means and SEMs of the Log_10_ (oocysts/gram fecal dry matter) (n = 7 for control; n = 6 for MMV665917 treated). Dashed line indicates the approximate qPCR assay limit of detection (LOD). **(B)** Scatter plot showing the area under the curve (AUC) for Log_10_ transformed fecal oocyst shedding for individual animals. The lines indicate the means and 95% confidence intervals. p value was determined using the unpaired, single-sided student’s t test.

We next tested the clinical and microbiologic efficacy of MMV665917 for treatment of cryptosporidiosis using the calf model. Neonatal bull calves were infected by oral administration of *C*. *parvum* Iowa isolate oocysts. The high inoculum used consistently resulted in infection and onset of severe diarrhea ~72 hours after administration (Figs 2A and 3A). Infected calves received either MMV665917 (22 mg/kg once daily for seven days) or vehicle alone (negative control) beginning on day four after infection, the time point corresponding to the second day of severe diarrhea. MMV665917 treatment reduced diarrhea severity within one day (Fig 2A), which corresponded to a two log reduction in the number of oocysts per gram of fecal dry matter within two days of beginning treatment (Fig 3A). Interestingly, low level oocyst shedding continued despite ongoing treatment and complete resolution of diarrhea. This was consistent with prior observations of the natural history of *Cryptosporidium* infection in dairy calves, which demonstrated persistent low level oocyst shedding after the resolution of illness (observations out to 28 days following infection; S1 Fig).

MMV665917 treatment reduced both the severity of diarrhea and the numbers of oocysts shed. The AUC for each animal calculated from a plot of the fecal consistency vs. time (including the first day of drug dosing) was used to quantify the magnitude of the reduction in diarrhea over the course of the study. Consistent with the general clinical observation of caregivers, MMV665917 treatment dramatically reduced the number of animal days with moderate-to-severe diarrhea (Fig 2B). Similarly, calculation of the AUC for Log_10_ transformed oocyst shedding by each animal demonstrated that MMV665917 treatment reduced overall shedding by ~94% (geometric mean of total oocyst shedding of 91,743 vs. 1,458,631 for treated vs. control), despite delaying treatment until the peak of diarrhea (Fig 3B).

**Fig 4:**
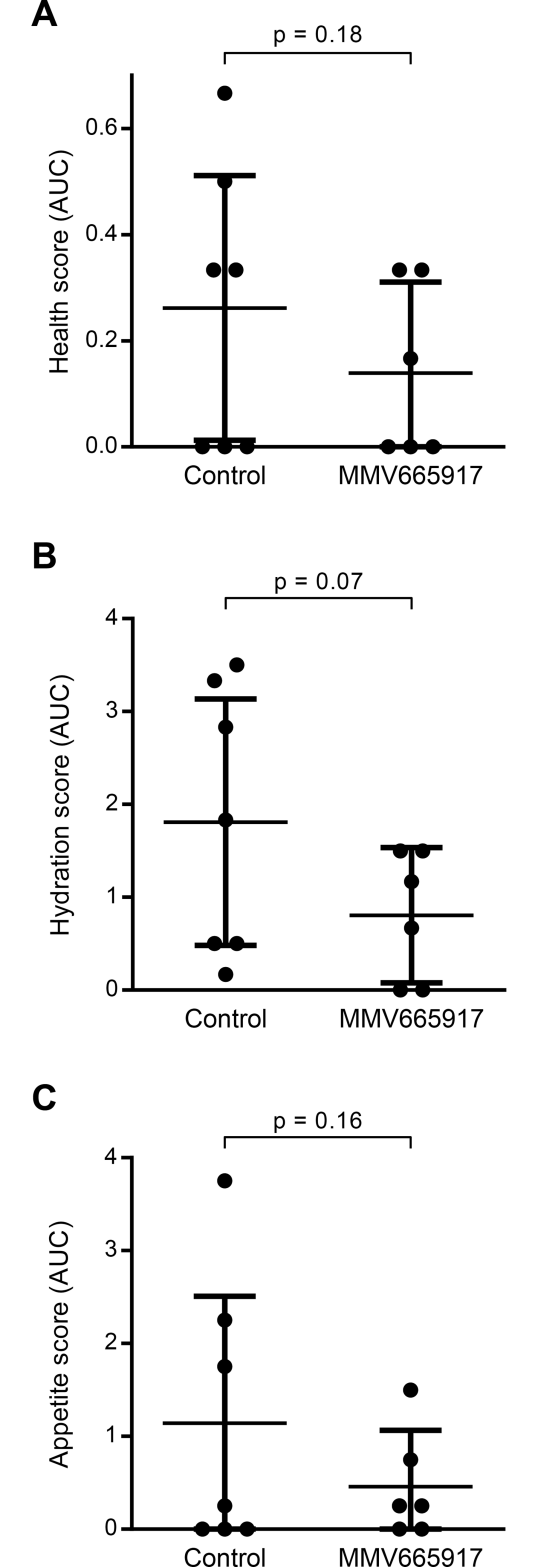
Effect of MMV665917 on secondary health measures in *C*. *parvum* infected calves. Overall health, hydration, and appetite were assessed twice daily on days 4–11 after infection using standardized three point scores (1 = normal, 3 = severely abnormal). The area under the curve (AUC) for each metric was determined for each animal’s average daily score plotted vs. time using a baseline score of 1 (normal). Each graph shows a scatter plot of data for individual animals, along with means and 95% confidence intervals (n = 7 for control; n = 6 for MMV665917 treated). p values were determined using the unpaired, single-sided student’s t test. **(A)** Overall health scores. **(B)** Hydration scores. **(C)** Appetite scores.

The effect of MMV665917 treatment on overall health, dehydration, and appetite of *C*. *parvum* infected calves was quantified using a standardized scoring system (Table 1) and calculation of the AUC for each parameter. All calves received aggressive supportive care in an attempt to mitigate unnecessary suffering, which limited the magnitude of differences that might be observed between the experimental groups. Nonetheless, there was a strong trend towards improved health with MMV665917 treatment as assessed by each of these secondary clinical outcome measures (Fig 4).

## Discussion

These studies validate the efficacy of the piperazine-based anti-*Cryptosporidium* drug lead MMV665917 in a clinical model of disease. This is a critical extension of previous mouse studies, because mice infected with *C*. *parvum* do not develop diarrhea. Dairy calves, on the other hand, develop a diarrheal illness very similar to that observed in infants following infection with *C*. *parvum* [14], one of the two main human pathogens. Our data show that MMV665917 is both clinically and microbiologically effective for treatment of cryptosporidiosis in new-born calves.

Since *C*. *parvum* is also an important pathogen of cattle, these data represent a key step for drug development directed towards treatment of both calves and people afflicted with cryptosporidiosis, and provide a strong rationale for further optimization of the piperazine-based MMV665917 chemotype. It was somewhat surprising that diarrhea did not reduce the fecal or serum concentrations of MMV665917 following oral administration, which may simplify dose optimization. Finally, it should be noted that the method used in this study of housing calves in confined pens is stressful to animals, and therefore, likely immunosuppressive [16], which further emphasizes the clinical efficacy of MMV665917.

This study has several limitations. Recently published target product profiles for anti-*Cryptosporidium* drugs specify a typical treatment duration of only 3 to 4 days for most patients (severely immunocompromised patients may require more prolonged treatment) [17, 18]. Thus, additional studies are needed to determine the minimum duration of MMV665917 that is efficacious. Also, only bulls were included due to limited access to heifers, so further studies are needed to address possible sex differences in MMV665917 efficacy. Despite prior studies demonstrating comparable *in vitro* potency against both *C*. *parvum* and the *C*. *hominis* TU502 isolate (Jumani, et al. Submitted manuscript), confirmation of *in vivo* efficacy against *C*. *hominis* is also needed, e.g. using the gnotobiotic piglet model [19]. Although no toxicity in animals has been observed, *in vitro* patch clamp studies demonstrated partial inhibition of the human ether-a-go-go (hERG) potassium channel by physiologically relevant concentrations of MMV665917, which indicates the possibility of cardiac toxicity. Additional safety studies are therefore needed to assess the possibility of cardiotoxicity, and medicinal chemistry optimization to increase potency and selectivity is likely warranted. Although the optimal pharmacokinetic properties for the MMV665917 series (e.g. high vs. low systemic bioavailability) remain to be determined, it is also possible that the hERG liability can be addressed by modifications that result in lower oral absorption. In any case, such medicinal chemistry optimization would likely be aided by knowledge of the molecular mechanism of MMV665917, which remains unknown for both *Cryptosporidium* and *Plasmodium* species. Nonetheless, based on its outstanding clinical and microbiologic efficacy and the relative ease of synthesizing it and related compounds, MMV665917 represents an excellent starting point for a full-fledged anti-*Cryptosporidium* drug lead optimization program.

## Supporting information

S1 Fig Natural history of *C*. *parvum* dairy calf infection using the protocol in this study. Calves were infected at 24–48 hours of age by oral administration of ~5 × 10^7^ *C*. *parvum* Iowa isolate oocysts, and fecal oocyst shedding was following using qPCR. The graph shows fecal oocyst shedding vs. time. Dashed line indicates the reliable limit of detection (LOD) for the qPCR assay. Data are the mean and SEM of the Log_10_ (oocysts/gram fecal dry matter) (n = 4 calves).
(EPS)
